# Increased systemic and adipose 11β-HSD1 activity in idiopathic intracranial hypertension

**DOI:** 10.1530/EJE-22-0108

**Published:** 2022-05-18

**Authors:** Connar S J Westgate, Keira Markey, James L Mitchell, Andreas Yiangou, Rishi Singhal, Paul Stewart, Jeremy W Tomlinson, Gareth G Lavery, Susan P Mollan, Alexandra J Sinclair

**Affiliations:** 1 Metabolic Neurology, Institute of Metabolism and Systems Research, College of Medical and Dental Sciences, University of Birmingham, Birmingham, UK; 2 Department of Neurology, University Hospitals Birmingham NHS Foundation Trust, Queen Elizabeth Hospital, Birmingham, UK; 3 Upper GI Unit and Minimally Invasive Unit, Birmingham Heartlands Hospital, University Hospitals Birmingham NHS Foundation Trust, Birmingham, UK; 4 Medical School, University of Leeds, Leeds, UK; 5 Oxford Centre for Diabetes, Endocrinology & Metabolism (OCDEM), NIHR Oxford Biomedical Research Centre, University of Oxford, Churchill Hospital, Headington, Oxford, UK; 6 Department of Biosciences, Nottingham Trent University, Nottingham, UK; 7 Birmingham Neuro-Ophthalmology, University Hospitals Birmingham NHS Foundation Trust, Queen Elizabeth Hospital, Birmingham, UK

## Abstract

**Context:**

Idiopathic intracranial hypertension (IIH) is a disease of raised intracranial pressure (ICP) of unknown etiology. Reductions in glucocorticoid metabolism are associated with improvements in IIH disease activity. The basal IIH glucocorticoid metabolism is yet to be assessed.

**Objective:**

The objective of this study was to determine the basal glucocorticoid phenotype in IIH and assess the effects of weight loss on the IIH glucocorticoid phenotype.

**Design:**

A retrospective case–control study and a separate exploratory analysis of a prospective randomized intervention study were carried out.

**Methods:**

The case–control study compared female IIH patients to BMI, age, and sex-matched controls. In the randomized intervention study, different IIH patients were randomized to either a community weight management intervention or bariatric surgery, with patients assessed at baseline and 12 months. Glucocorticoid levels were determined utilizing 24-h urinary steroid profiles alongside the measurement of adipose tissue 11β-HSD1 activity.

**Results:**

Compared to control subjects, patients with active IIH had increased systemic 11β-hydroxysteroid dehydrogenase (11β-HSD1) and 5α-reductase activity. The intervention study demonstrated that weight loss following bariatric surgery reduced systemic 11β-HSD1 and 5α-reductase activity. Reductions in these were associated with reduced ICP. Subcutaneous adipose tissue explants demonstrated elevated 11β-HSD1 activity compared to samples from matched controls.

**Conclusion:**

The study demonstrates that in IIH, there is a phenotype of elevated systemic and adipose 11β-HSD1 activity in excess to that mediated by obesity. Bariatric surgery to induce weight loss was associated with reductions in 11β-HSD1 activity and decreased ICP. These data reflect new insights into the IIH phenotype and further point toward metabolic dysregulation as a feature of IIH.

## Introduction

Idiopathic intracranial hypertension (IIH) is a disease characterized by raised intracranial pressure (ICP), papilledema, and disabling headaches (1, 2). IIH predominantly affects obese women of the reproductive age, and its incidence is increasing in parallel with the obesity epidemic (3, 4). Disease modification of IIH is through weight management, and the IIH weight trial (IIH:WT) provided evidence that weight loss through bariatric surgery inferred long-term remission of ICP as compared to a community weight management (CWM) intervention (5, 6, 7).

Defining the pathophysiology of IIH and identifying biomarkers to guide diagnosis and target management were deemed top priorities for research by both healthcare professionals and people with IIH in a priority-setting partnership (8). IIH has long been thought to be a disease isolated to the CNS; however, recent evidence has demonstrated systemic metabolic features linked to but in excess of those seen in simple obesity (9, 10, 11). IIH patients have truncal obesity, an increased risk of cardiovascular disease and type 2 diabetes mellitus in addition to being more insulin resistant and having altered adipose tissue function with a greater magnitude of derangement than that mediated by obesity (10, 12, 13). Additionally, IIH patients have a unique phenotype of serum and cerebrospinal fluid (CSF) androgen excess, highlighting altered steroid metabolism (9).

Glucocorticoids (GC) and 11β-hydroxysteroid dehydrogenase (11β-HSD) are associated with disease activity in IIH. Pre-receptor corticosteroid availability is mediated by the bidirectional enzyme, 11β-HSD (14). This enzyme has two isoforms: 11β-HSD1 which acts predominantly as an oxoreductase and activates cortisol from cortisone and 11β-HSD2, which inactivates cortisol to cortisone. 11β-HSD1 is expressed in a wide variety of tissues but its principal role in humans is mediating local cortisol availability where, for example, in the adipose tissue, it can promote adipocyte differentiation and drive hepatic glucose output (15). In IIH, 11β-HSD1 activity is decreased in association with a reduction in ICP following a therapeutic diet (16). Change in systemic 11β-HSD1 activity correlates significantly with change in ICP. A randomized controlled trial in IIH demonstrated that specific therapeutic inhibition of 11β-HSD1 (AZD4017) reduces ICP (17). Cognitive impairment has been associated with GC excess (18, 19). Cognitive dysfunction is documented in patients with IIH and has been shown to significantly correlate with serum cortisol levels, a known driver of cognitive impairment (20, 21). Normalizing serum cortisol through weight loss improves the cognitive impairment in IIH (6, 20).

While these data suggest that cortisol secretion and its metabolism by 11β-HSD1 activity are relevant in IIH etiology, it is unknown if IIH patients have altered systemic 11β-HSD1 activity compared to weight-matched controls. Indeed, in there is no consensus on what simple obesity does to systemic 11β-HSD1 activity (15, 22, 23, 24). Furthermore, it is unclear what may be driving altered 11β-HSD1 activity in IIH.

Here, the urinary GC phenotype in IIH is defined, the role of bariatric surgery on the urinary GC phenotype is assessed, and the contribution of adipose tissue to the IIH GC phenotype is understood.

## Methods

### Study conduct

IIH subjects were identified from multiple UK centres, and samples were collected following informed, written consent. The trials received ethical approval for the IIH and control cohorts from the York and Humber-Leeds West Research Ethic committee (REC) (13/YH/0366), Dudley local REC (06/Q2702/64), and the Black Country REC (14/WM/0011) (collected as part of three separate ethical applications).

Control patients were recruited via advertisement, where sample collection occurred following informed written consent. Sample collection was approved by the South Birmingham Local REC and the Black Country REC (14/WM/0011). Control patients for adipose tissue experiments were recruited from elective National Health Service bariatric surgery procedures following written informed consent and was approved by the Black Country REC (14/WM/0011).

### Study population

Adult (18–55 years) female IIH patients with active IIH (papilloedema ≥Frisén grade 1 and lumbar puncture opening pressure (LP OP) ≥ 25 centimeters of cerebrospinal fluid (cmCSF) on the date of research assessment visit) diagnosed in line with the International IIH Guideline criteria were recruited (5). IIH patients at any stage of their active disease were included. Control patients met the same inclusion criteria as the IIH patients, with the absence of an IIH diagnosis.

### Exclusion criteria

Exclusion criteria for all patients included receiving hormone manipulating medication (including contraceptives and those exposed to GC therapy in the last 3 months), significant comorbidities including known endocrinopathies, and the inability to give informed consent. Additionally, IIH patients were excluded if they were pregnant during the visit.

### Assessments

All participants underwent detailed medical history and examination. Anthropometric data were recorded. Lumbar punctures were carried out in all IIH patients and conducted in the left lateral decubitus position with knees bent at a 90° angle or more and (LP OP) recorded. Twenty-four-hour urine samples for urinary steroid metabolite profiling were also provided by patients in attendance at the research facility. Urine samples were stored at −80°C and analyzed after a maximum of one freeze––thaw cycle. Patients were fasted overnight for all visits.

### Case–control study

A case–control study comparing IIH with matched controls was conducted to determine the urinary GC metabolome. The control subject cohort was group matched (retrospectively) to the IIH population for age, gender, and BMI.

### Weight loss study

This was a sub-study of the IIH:WT (a randomized controlled trial comparing CWM intervention to bariatric surgery in IIH), the full protocol and primary results have been published elsewhere (NCT02124486) (6, 25). Patients were randomized 1:1 to either a CWM intervention (utilizing weight watchers) or a bariatric surgery program (laparoscopic adjustable gastric banding, Roux-en-Y gastric bypass or laparoscopic sleeve gastrectomy). Randomization was done by a computer-generated randomization list via a phone line at the Birmingham Clinical Trials Unit. Patient visits occurred at baseline and 12 months after baseline as per the previously published protocol (25). This sub-study evaluated the steroid metabolome changes in 24-h urine samples collected at baseline and 12 months after baseline, following intervention. Patients randomized to surgery had a median time from randomization to bariatric surgery of 4.4 months (range, 2.2–10.3 months).

### Urinary steroid profiling

Systemic steroid metabolism in 24-h urine samples was profiled using gas chromatography–mass spectrometry and liquid chromatography–mass spectrometry (LC-MS) as previously described (26). 11β-HSD1 activity was assessed using the ratio of the tetrahydrometabolites of cortisol (5α-THF+THF) to tetrahydrocortisone (THE). 11β-HSD2 activity was assessed via the free urinary cortisol to cortisone ratio (23). Total GC metabolite excretion was assessed as the sum of 5α-THF + THF + THE + cortolones + cortols + cortisol +  cortisone and provided an accurate marker of 24-h cortisol secretion rate (23).

### HOMA2-IR assessment

Fasting insulin was measured using commercially available assays (Mercodia), according to the manufacturer’s instructions. Homeostatic model assessment (HOMA2)-IR was calculated using the program HOMA calculator v2.2.3 (https://www.dtu.ox.ac.uk/homacalculator/).

### Leptin ELISA

Leptin was quantified in serum using the human leptin DuoSet ELISA (DY-398, Bio-Techne, Minneapolis, MN, USA). The ELISA was carried out according to the manufacturer’s instructions using the recommended ancillary kit (Bio-Techne, DY008). Serum was diluted 1:100 in reagent diluent. Samples were run in duplicate.

### Interleukin 6 ELISA

Interleukin 6 (IL-6) was quantified in serum by ELISA as per manufacturer's instructions using the Human IL-6 DuoSet ELISA (R&D Systems, Cat no. DY206, UK). The ELISA was carried out according to the manufacturer’s instructions using the recommended ancillary kit (Bio-Techne, DY008). Samples were run in duplicate.

### Adipose tissue collection

At baseline, subcutaneous adipose tissue was collected from IIH and control subjects following an overnight fast (from midnight). Subcutaneous adipose tissue was biopsied and either placed immediately in RNA later or into phenol-free DMEM/F12 (Thermofisher), with no antibiotics.

### *Ex vivo* adipose 11β-hydroxysteroid dehydrogenase activity

Subcutaneous abdominal adipose tissue explants (~100 mg, tested in triplicate) were incubated in phenol-free DMEM/F12 containing 100 nM cortisone for 24 h in glass tubes. Control experiments were also conducted with identical conditions but no adipose tissue. Steroid conversion was quantified using LC-MS as previously described and normalized to explant mass (27, 28).

### Statistical analysis

Statistical analysis was performed using Graphpad Prism 9 (Graphpad Software Inc). Data are presented as mean ± s.d. unless otherwise stated. Data normality was assessed by a Shapiro–Wilk normality test. Where data were normally distributed, unpaired two-tailed *t*-tests (equal variance) or Welch’s test (unequal variance) were employed, whereas non-parametric data were assessed via Mann–Whitney *U*-test. For multigroup comparisons, two-way repeated-measures ANOVA followed by Sidak’s multiple comparisons test were utilized. Spearman’s rank correlation coefficient (ρ) and Pearson’s correlation coefficient (r) were used for assessing correlations in the IIH cohorts. Where data points are missing, data were not imputed. We did not correct for multiple comparisons as this would have increased the likelihood of type II errors. Results were judged significant at *P* < 0.05.

## Results

### Patient characteristics

Controls (*n* = 17) and IIH (*n* = 27) patients (all female) were matched for age (41.7 ± 4.2 vs 39.4 ± 5.9 years, *P* = 0.18) and BMI (34.9 ± 3.8 vs 38.4 ± 8.4 kg/m^2^, *P* = 0.1). IIH patients had a mean ICP of 35.1 ± 4.6 cmCSF (Table 1). There was no difference in the degree of insulin resistance between the groups (HOMA2-IR: 1.7 ± 1.1 vs 1.8 ± 2.0, *P* = 0.8) (Table 1).

**Table 1:** Urinary steroid profiling characteristics in control vs IIH. Data are presented as mean ± s.d.

Characteristics	Control (*n* = 17)	IIH (*n* = 27)
Age (years)	41.7 ± 4.2	39.5 ± 6.0
BMI (kg/m^2^)	34.8 ± 3.8	38.4 ± 8.4
ICP (cm CSF)	N/A	40.6 ± 3.7
Sex (female, %)	100%	100%
Fasting insulin (mIU/L)	13.5 ± 9.6	13.39 ± 7.6
HOMA2-IR	1.7 ± 1.1	1.8 ± 2.0
Urine (µg/24 h)		
5α-THF	1177 ± 681	1421 ± 954
THF	1472 ± 817	1331 ± 597
THE	2960 ± 1581	2662 ± 1418
Cortisol (F)	46 ± 20	71 ± 36**
Cortisone (E)	85 ± 32	107 ± 60
Total GC metabolites	8532 ± 4068	8039 ± 3807
Derivative measurements		
(5α-THF+THF/THE)	0.9 1 ±0.1	1.13 ± 0.34*
F/E	0.50 ± 0.16	0.53 ± 0.14
5α-THF/THF	0.84 ± 0.33	1.20 ± 0.69*

*P < 0.05, **P < 0.01.

cmCSF, centimetres of cerebrospinal fluid; GC, glucocorticoid; HOMA, homeostatic model assessment; ICP, intracranial pressure; THE, tetrahydrocortisone; THF, tetrahydrocortisol; 5α-THF, 5α-tetrahydrocortisol.

### Basal glucocorticoid metabolome in IIH

**Figure 1:**
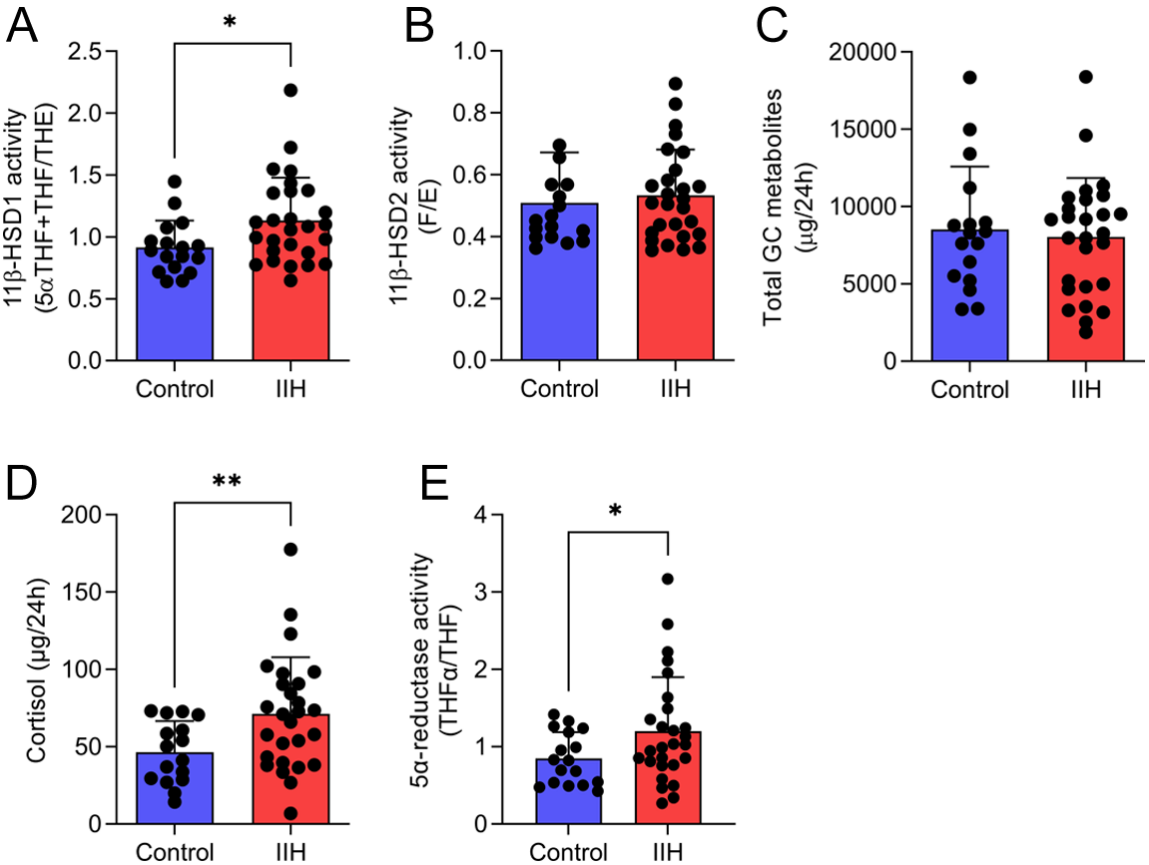
Altered glucocorticoid metabolism in patients with IIH. Twenty-four-hour urine steroid metabolomic assessment in control (17) and IIH patients (27). (A) 11β-HSD1 activity was denoted by 5α-THF+THF/THE. (B) 11β-HSD2 activity was denoted by urinary free cortisol/cortisone (F/E). (C) Total glucocorticoid metabolite excretion. (D) Cortisol secretion. (E) 5α-reductase activity. Data are presented as mean ± s.d., Mann–Whitney *U*-test for A and B, *t*-test for (C) and *t*-test with Welch’s correction for (D) and (E). *P < 0.05, **P < 0.01. IIH, idiopathic intracranial hypertension; 11β-HSD1, 11β-hydroxysteroid dehydrogenase; 5α-THF+THF/THE, 5α-tetrahydocortisol+tetrahydrocortisol/tetrahydrocortisone. A full color version of this figure is available at https://doi.org/10.1530/EJE-22-0108.

We sought to investigate the systemic steroid metabolome in IIH patients using 24-h urine collections. Mass spectrometry analysis revealed a significant increase in systemic 11β-HSD1 activity in IIH (IIH 1.131 ± 0.34 vs control 0.91 ± 0.21 THF+THFα/THE; *P* = 0.019, Fig. 1A). Linked to this, IIH patients had increased urinary cortisol levels (71.2 ± 36.8 vs 46.5 ± 20.1 µg/24 h, *P* = 0.0069, Fig. 1D). Markers of 11β-HSD2 activity (Fig. 1B) and total GC excretion were not altered (Fig. 1C). As inferred from the urinary 5α-THF/THF ratio, we also demonstrated increased activity of the cortisol and testosterone metabolizing enzyme 5α-reductase in IIH (0.84 ± 0.33 vs 1.20 ± 0.69, *P* = 0.03) (Fig. 1E). All other measured steroids were found to be unchanged, with the exception of pregnanediol which was increased in IIH patients (391.1 ± 290.2 vs 155.5 ± 108.9 µg/24 h, *P* = 0.0018) (Supplementary Table 1, see section on supplementary materials given at the end of this article).

Markers of 11β-HSD1 activity (r = −0.13, *P* = 0.4) and 5α-reductase activity (r = −0.11, *P* = 0.59) did not correlate with ICP at baseline. Additionally, we demonstrated no correlation between 11β-HSD1 activity and BMI (ρ = −0.1, *P* = 0.6), fasting insulin (ρ = 0.29, *P* = 0.16) or HOMA2-IR (ρ = 0.22, *P* = 0.29). We observed no correlation between 5α-reductase activity and BMI (ρ = −0.25, *P* = 0.2), fasting insulin (ρ = 0.06, *P* = 0.77), and HOMA2-IR (ρ = 0.02, *P* = 0.87).

### Weight loss study

The IIH:WT established that weight loss following bariatric surgery led to significant sustained reduction in ICP in IIH (6). In this sub-study, 24-h urine samples were collected at baseline and at 12 months (16 from the CWM intervention and 13 from the bariatric surgery arm). Among these participants at baseline, both IIH cohorts were well matched for gender (100% female), BMI (44.7 ± 8.2 vs 44.0 ± 7.3 kg/m^2^, *P* = 0.79), age (31.9 ± 7.9 vs 32.0 ± 7.5 years, *P* = 0.79), and ICP (32.6 ± 4.8 vs 35.0 ± 5.1 cmCSF, *P* = 0.18).

Twelve months following enrollment, the participants randomized to surgery had a lower BMI (44.1 ± 8.8 vs 34.8 ± 8.0 kg/m^2^, *P* = 0.007) and had a lower ICP (30.4 ± 5.6 vs 23.5 ± 6.6, *P* = 0.008) compared to participants randomized to the CWM intervention (Table 2).

**Table 2:** Urinary steroid profiling characteristics table following intervention. Data are presented as mean ± s.d.. There was no statistical significance between CWM and surgery at baseline.

	Baseline		12 Months	
	CWM (16)	Surgery (13)	CWM (16)	Surgery (13)
Age (years)	31.8 ± 7.9	32.6 ± 7.4		
BMI (kg/m^2^)	44.7 ± 8.1	44.0 ± 7.6	44.1 ± 8.8	34.0 ± 8.0**^,####^
ICP (cmCSF)	32.6 ± 4.8	35.2 ± 5.1	30.3 ± 5.6	23.6 ± 6.6**^,####^
Sex (female, %)	100%	100%		
Fasting insulin (mIU/L)	17.4 ± 7.6	10.7 ± 3.8	14.7 ± 6.1^$$^	4.3 ± 4.3*^,###^
HOMA2-IR	1.9 ± 0.8	1.5 ± 0.6	1.1 ± 0.4 ^$$^	0.4 ± 0.4*^, ###^
Leptin (ng/mL)	89.3 ± 37.0	76.8 ± 32.2	79.3 ± 38.8	31.9 ± 24.0***^, ####^
IL-6 (pg/mL)	5.8 ± 2.7	6.3 ± 2.1	5.4 ± 1.9	5.0 ± 2.1
Urine (µg/24 h)				
5α-THF	518 ± 537	324 ± 304	897 ± 641^$^	403 ± 304*
THF	2305 ± 1708	2058 ± 1406	2640 ± 1309	1967 ± 899
THE	3435 ± 2121	2666 ± 1741	5055 ± 2231^$^	3780 ± 1836
Cortisol (F)	69 ± 51	63 ± 47	65 ± 27	66 ± 25
Cortisone (E)	90 ± 70	73 ± 41	118 ± 52	106 ± 55
Total GC metabolites	10057 ± 5881	7502 ± 4806	14138 ± 6597	12195 ± 6026
Derivative measurements				
5α-THF+THF/THE	0.799 ± 0.14	0.933 ± 0.22	0.699 ± 0.13	0.660 ± 0.16^####^
F/E	0.812 ± 0.56	0.816 ± 0.24	0.563 ± 0.11^$$$$^	0.683 ± 0.22^#^
5α-THF/THF	0.231 ± 0.10	0.188 ± 0.14	0.32 ± 0.13^$$^	0.22 ± 0.09

*Statistical comparisons between CWM and surgery at 12 months; ^#^Statistical comparison between surgery baseline and surgery 12 months; ^$^Statistical comparison between CWM baseline and surgery 12 months; *P < 0.05; **P < 0.01; ***P < 0.001; ****P < 0.0001.

cmCSF, centimeters of cerebrospinal fluid; GC, glucocorticoid; HOMA, homeostatic model assessment; ICP, intracranial pressure; IL-6, interleukin 6; THE, tetrahydrocortisone; THF, tetrahydrocortisol; 5α-THF, 5α-tetrahydrocortisol.

**Figure 2:**
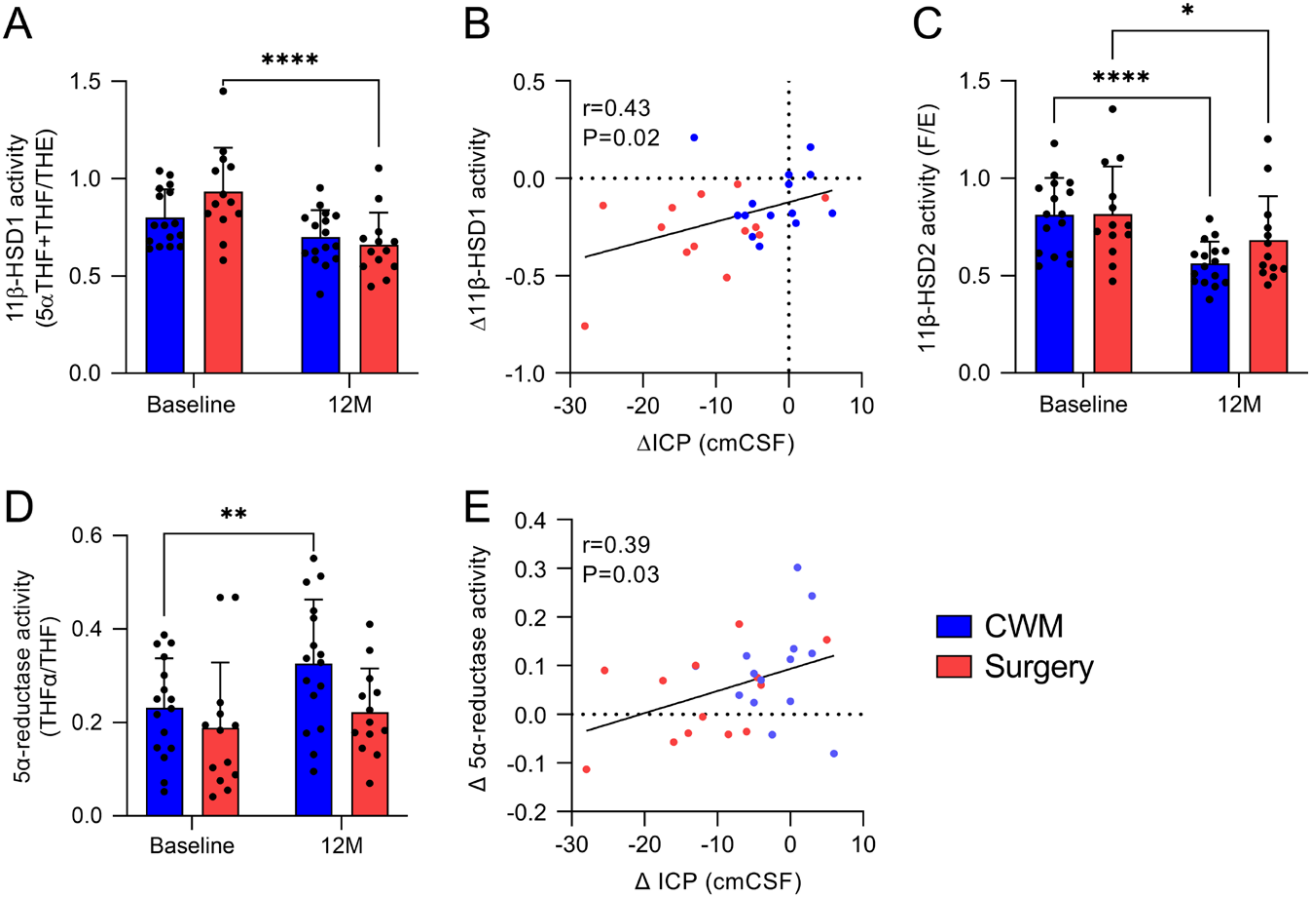
IIH urinary steroid metabolome following weight loss. Twenty-four-hour urine steroid metabolome profiling at baseline and 12 months in diet (16) and surgical (13) IIH patients. (A) Change in 11β-HSD1, (B), scatter graph of change in 11β-HSD1 activity vs change in intracranial pressure (ICP), (C) change in 11β-HSD2 activity, (D) change in 5α-reductase activity, and (E) scatter graph of change in 11β-HSD1 activity vs change in ICP. Two-way repeated measures ANOVA followed by Sidak’s multiple comparisons test for (A), (B), and (D). Pearson’s correlation for (B) and (E). Data are presented as mean ± s.d. *P < 0.05. cmCSF, centimetres of cerebrospinal fluid; IIH, idiopathic intracranial hypertension; 11β-HSD1, 11β-hydroxysteroid dehydrogenase. A full color version of this figure is available at https://doi.org/10.1530/EJE-22-0108.

In the bariatric surgery cohort, 11β-HSD1 activity was significantly reduced at 12 months compared to baseline (0.93 ± 0.2 vs 0.66 ± 0.1, *P* < 0.0001, Fig. 2A) while there was no significant reduction in the CWM arm. At 12 months, the change (Δ) in 11β-HSD1 activity from baseline was significantly greater in the bariatric surgery cohort compared to those in the CWM arm (−0.1 ± 0.15 vs −0.27 ± 0.19 Δ11β-HSD1 activity, *P* = 0.013, Supplementary Table 2). The change in 11β-HSD1 activity was significantly associated with change in ICP in all groups (r = 0.43, *P* = 0.02, Fig. 2B). 11β-HSD2 activity was reduced following bariatric surgery (0.82 ± 0.2 vs 0.68 ± 0.2, *P* = 0.013) and within the CWM arm (0.81 ± 0.2 vs 0.56 ± 0.1, *P* < 0.0001, Fig. 2C) with no difference between arms at 12 months (−0.25 ± 0.12 vs −0.13 ± 0.21 Δ11β-HSD2, *P* = 0.09, Supplementary Table 2). 5α-reductase activity remained unchanged at 12 months in the bariatric surgery group (0.18 ± 0.1 vs 0.22 ± 0.1, *P* = 0.39); however, the activity was noted to be increased in the CWM arm (0.23 ± 0.1 vs 0.32 ± 0.13, *P* = 0.0012, Fig. 2D) but there was no difference in the change at 12 months (0.09 ± 0.1 vs 0.03 ± 0.08 Δ5α-reductase activity, *P* = 0.1, Supplementary Table 2). Change in 5α-reductase activity was correlated positively with change in ICP (r = 0.39, *P* = 0.03, Fig. 2E). Changes in other analyzed steroids can be found in Supplementary Table 2.

The relationship of 11β-HSD1 and 5α-reductase activity to other markers of metabolic dysregulation and inflammation was then evaluated. The change in systemic 11β-HSD1 activity had a trend to correlation with change in fasting leptin (ρ = 0.37, *P* = 0.05), fasting insulin (ρ = 0.38, *P* = 0.085) and HOMA2-IR (ρ = 0.404, *P* = 0.07), with no correlation with change in the inflammation marker IL-6 (ρ = −0.004, *P* = 0.98). The change in 5α-reductase activity was not associated with changes in leptin, fasting insulin, HOMA2-IR, or IL-6.

### Subcutaneous adipose 11β-HSD1 activity

**Figure 3:**
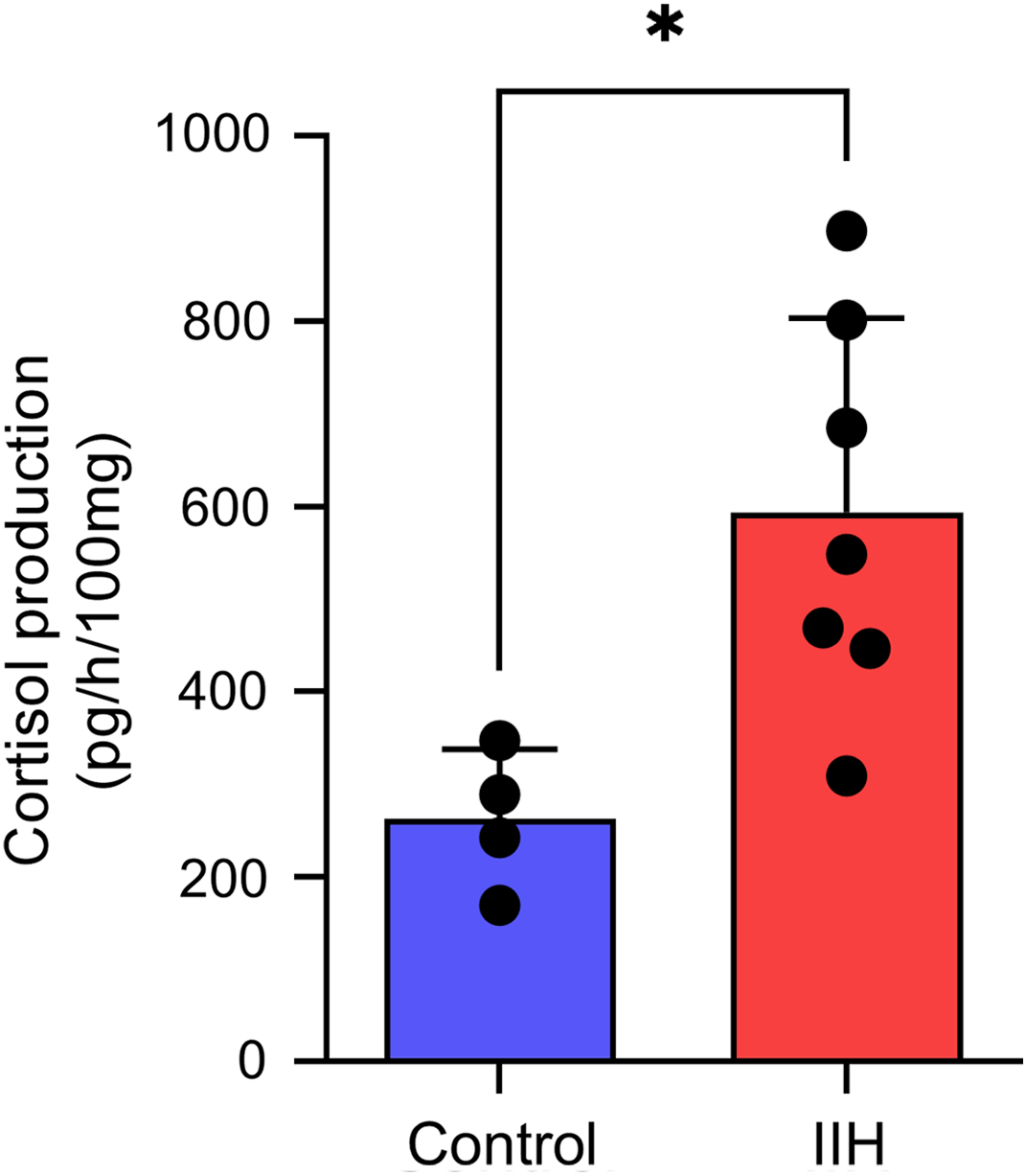
Increased 11β-HSD1 activity in subcutaneous adipose tissue explants from patients with IIH and controls. 11β-HSD1 activity as measured by cortisol production in control (4) and IIH (7) subcutaneous adipose explants (unpaired *t*-test). Data is presented as mean ± s.d.. *P < 0.05. IIH, idiopathic intracranial hypertension; 11β-HSD1, 11β-hydroxysteroid dehydrogenase. A full color version of this figure is available at https://doi.org/10.1530/EJE-22-0108.

We have shown increased systemic 11β-HSD1 activity in IIH reduced in parallel with decreased ICP in the context of weight loss. Next, we assessed the ability of 11β-HSD1 to generate cortisol in subcutaneous adipose tissue from IIH patients. We demonstrated a 2.2-fold increase in 11β-HSD1 activity in IIH subcutaneous adipose explants when compared to control participants (IIH 593.8 ± 79.3 vs control 261.9 ± 37.6 pg cortisol/h/100 mg; *P* = 0.015, Fig. 3).

## Discussion

IIH has traditionally been regarded as a disease of the neuro-ophthalmic axis (29). The link to obesity is long established but data are emerging identifying systemic metabolic perturbations in excess to that driven by simple obesity alone (7, 10, 11). Inhibition of 11β-HSD1 in IIH has been demonstrated to have therapeutic benefits including reduction of ICP, improved lipid profiles (decreased cholesterol, increased HDL, and cholesterol/HDL ratio), decreased markers of hepatic dysfunction, and increased lean muscle mass (17, 30). However, it is unknown if IIH patients have deranged GC metabolism. In this study, we conducted GC phenotyping in patients with IIH and then evaluated the impact of therapeutic weight loss on GC metabolism in IIH.

We have demonstrated increased urinary cortisol and urinary systemic 11β-HSD1 activity in IIH compared to matched controls with obesity. 11β-HSD1 activity has been previously shown to be modified in the context of obesity. There is no clear consensus on what happens to systemic 11β-HSD1 activity, where multiple studies report differing direction of change or no change at all (15, 22, 23, 24). However, it is clear that hepatic 11β-HSD1 activity is reduced in the context of obesity, where it is the main contributor to systemic 11β-HSD1 activity (31, 32, 33, 34, 35). In contrast, subcutaneous adipose 11β-HSD1 activity is increased in the context of obesity (22, 24). Our data suggest that systemic 11β-HSD1 activity in IIH is greater than in obesity alone. Thus, irrespective of the change of systemic 11β-HSD1 in simple obesity, IIH likely represents a novel 11β-HSD1 phenotype distinct to obesity. We did not compare IIH patients to lean controls, as such this could be an avenue of future investigation.

A number of historical case reports have linked IIH to GC therapy but GCs have in the past been prescribed to ameliorate IIH (36). Hence, the interrelationship between IIH and iatrogenic GCs is unclear but 11β-HSD1 maybe relevant (15). The regulation of 11β-HSD1 activity is known to be tissue-, gender-, and species-specific (35). Importantly, GCs themselves can regulate 11β-HSD1 activity (35). Cortisol, dexamethasone, and prednisolone have been noted to both activate and suppress 11β-HSD1 activity depending on the tissue studied (33, 37). The role of GCs in regulating 11β-HSD1 activity in the choroid plexus, the tissue that regulates ICP through CSF secretion, has not been evaluated. GCs are no longer routinely used in the management of IIH due to lack of evidence of efficacy and the significant risk of weight gain exacerbating the underlying disease (5, 38).

These data have also demonstrated increased systemic 5α-reductase activity in IIH. 5α-reductase is a pivotal enzyme involved in the breakdown of cortisol and conversion of testosterone to dihydrotestosterone with established sexual dimorphism (higher levels in females) demonstrated in rodents (39). 5α-reductase activity is enhanced in obesity and specifically in the adipose tissue (31). Here, we have illustrated increased systemic 5α-reductase activity in IIH compared to gender and BMI-matched controls. We have previously identified increased systemic and CSF testosterone in IIH (9). In this setting, the elevated 5α-reductase activity may reflect a compensatory mechanism to breakdown the excess cortisol in IIH. This may consequently increase the activation of testosterone to dihydrotestosterone, where androgens are potentially linked to the raised ICP in IIH (9).

To investigate whether weight loss improves the GC phenotype in IIH, the effects of bariatric surgery vs CWM were compared. The IIH:WT demonstrated that surgically mediated weight loss reduced systemic 11β-HSD1 activity; these results being similar to a previous very low-calorie diet study (16). This is, however, in contrast to a study on non-IIH obese individuals where bariatric surgery did not alter systemic 11β-HSD1 activity, despite weight loss (40). This could be potentially explained by the differential baseline hormonal and metabolic phenotype seen in IIH, as compared to those with obesity (but without IIH). Further evidence was provided when the 11β-HSD1 inhibitor AZD4017 was evaluated in a randomized control trial which reported a reduction in ICP in those with active IIH (17). *In vivo* studies have demonstrated that hydrocortisone increases CSF secretion in obese rats, suggesting a direct effect of active GCs on ICP dynamics (41). Indeed, we report that change in 11β-HSD1 activity correlates with change in ICP, further linking GCs to ICP levels. Together, our data and that of others suggest that 11β-HSD1 activity is likely linked to the raised ICP in IIH, although the potential mechanism underlying this requires further investigation.

Our findings of increased systemic 11β-HSD1 activity and the subsequent reduction in 11β-HSD1 activity with weight loss could be linked to the findings that patients with IIH have cognitive deficits that improve with weight loss (20). Within the CNS, 11β-HSD1 is involved in mediating mood and memory. An increase in cortisol exposure has been linked to cognitive decline and Alzheimer’s disease (42). Thus, it is interesting that in patients with IIH, cognitive improvement has been previously associated with decreased systemic 11β-HSD1 activity (20).

The association between weight loss and reduction in systemic 11β-HSD1 activity suggests that adipose tissue could be contributing to the increased 11β-HSD1 activity. Here, we demonstrated a doubling of subcutaneous adipose tissue 11β-HSD1 activity in IIH. Given that obesity confers an increase in adipose 11β-HSD1 activity, this increase in IIH is in excess to that driven by obesity alone (24). IIH adipose tissue has been previously demonstrated to have phenotypic features of GC excess, including depletion of ribosomal subunits, increased leptin secretion, and increased lipid turnover (10). Consequently, the current findings increased adipose 11β-HSD1 activity provides an explanation for the IIH adipose phenotype. It is already known that people with IIH have increased abdominal obesity, and with our demonstration of increased subcutaneous adipose 11β-HSD1 activity, it is therefore possible that subcutaneous adipose in IIH is directly contributing to the increased systemic measurement of 11β-HSD1 activity due to the compounding effects of increased 11β-HSD1 activity in a tissue that is more abundant in IIH (10, 13). This may also explain why other markers of GC metabolism remain unaltered in IIH. This is similar to rheumatoid arthritis, where tissue level 11β-HSD1 activity increases contribute to increased systemic 11β-HSD1 activity (43, 44). However, it is likely that there are other tissues contributing to increased systemic 11β-HSD1 that were not assessed in the present study. Indeed, although the contribution of subcutaneous adipose tissue to systemic 11β-HSD1 is unknown, visceral adipose is a major contributor and thus could be a focus of further research (45).

It has previously been demonstrated that inflammation increases 11β-HSD1 activity and thus could be a factor in IIH 11β-HSD1 increases. Previous studies demonstrated that IIH patients have unaltered circulating level of the pro-inflammatory cytokine IL-6 compared to obese controls (20, 46). Moreover, we demonstrate the IL-6 is unaltered following weight loss in this study. However, other pro-inflammatory cytokines which also can increase 11β-HSD1 activity such as IL-1B, IL-8, and TNF-α are increased in IIH suggesting that inflammation could play a role (46). However, this has not been assessed in the adipose of IIH patients. Consequently, the mechanisms underlying the increased subcutaneous adipose 11β-HSD1 activity remain unelucidated and should be a focus of further research.

IIH is a rare disease, and consequently modest numbers of IIH patients were utilized in the present study (3, 4, 12). However, our IIH patients were meticulously phenotyped, thus this cohort is representative of female IIH patients with active disease. We also acknowledge that the control cohort was small due to finding an obesity-matched population with no comorbidities was challenging; however, the controls recruited to this study were valuable as they enabled us to infer biological insights into IIH. Adipose samples were not sought following weight loss due to ethical considerations. Therefore, we were not able determine if weight loss reduced the 11β-HSD1 activity of the adipose; however, this study represents the first demonstration of 11β-HSD1 activity in IIH adipose tissue (17).

Patients randomized to surgery had different surgical interventions based on clinical need (25). Each surgical type has been suggested to have subtle difference in their effect on the hypothalamic–pituitary–adrenal axis and thus on potential GC phenotype (47). However, this study was not powered to determine the effect of a particular surgical intervention on the GC phenotype. We did not assess the hepatic 11β-HSD1 activity in this study, although this has been evaluated previously in IIH, where hepatic 11β-HSD1 activity did not correlate with ICP (17). Future studies assessing hepatic 11β-HSD1 in IIH patients vs controls are warranted.

## Conclusions

In summary, this study provides evidence that GC metabolism is dysregulated in IIH with 11β-HSD1 activity increased both systemically and within the subcutaneous adipose tissue. Therapeutic weight loss in IIH led to systemic reduction in the cortisol-metabolizing enzymes, 11β-HSD1 and 5α-reductase. The reduction in both 11β-HSD1 and 5α-reductase activity was associated with falling ICP levels suggesting a potential link to disease pathogenesis. It remains unclear if the altered GC phenotype in IIH is directly driving ICP dysregulation or a marker of systemic metabolic dysregulation.

## Supplementary Material

Supplementary Material

## Declaration of interest

SPM reports consulting and advisory board fees from Invex Therapeutics, teaching honoria from Heidelberg engineering during the conduct of the study; teaching honoria from Chugai-Roche Ltd, Allergan, Santen, Roche; advisory board fees from Janssen; consultancy fees from Neurodiem, outside the submitted work. A Y reports receiving speaker fees from Teva, UK outside the submitted work. A J S reports personal fees from Invex therapeutics, during the conduct of the study. J W T has received consultancy fees from Lumos and research funding from AstraZeneca. All other authors declare no competing interests.

## Funding

A J S was funded by a National Institute for Health Research http://dx.doi.org/10.13039/100005622 (NIHR) clinician scientist fellowship (NIHR-CS-011-028) and the Medical Research Council http://dx.doi.org/10.13039/501100000265, UK (MR/K015184/1) for the duration of the study. A J S is funded by a Sir Jules Thorn Award for Biomedical Science. The trial was funded through the Medical Research Council http://dx.doi.org/10.13039/501100000265 Asset Sharing Scheme, UK (MR/K015184/1). A J S is funded by a National Institute for Health Research http://dx.doi.org/10.13039/100005622 (NIHR) Clinician Scientist Fellowship (NIHR-CS-011-028). G G L is supported by a Wellcome Trust http://dx.doi.org/10.13039/100010269 Senior Research Fellowship (104612/Z/14/Z). J W T is supported by MRC program (MR/P011462/1) and by the Oxford NIHR Biomedical Research Centre. The views expressed are those of the authors and not necessarily those of the UK National Health Service, the NIHR, or the UK department of Health and Social Care. Data from IIH:WT (trial beginning: July 25, 2014; date of registration: April 28, 2014; ClinicalTrials.gov: NCT02124486) and from IIH Drug trial (trial beginning: April 25, 2014; date of registration: December 20, 2013; ClinicalTrials.gov: NCT02017444).

## Data availability statement

Anonymized individual participant data will be made available along with the trial protocol and statistical analysis plan. Proposals should be made to the corresponding author and will be reviewed by the Birmingham Clinical Trials Unit Data Sharing Committee in discussion with the Chief Investigator. A formal Data Sharing Agreement may be required between respective organisations once release of the data is approved and before data can be released. Restrictions apply to the availability of some or all data generated or analyzed during this study to preserve patient confidentiality or because they were used under license. The corresponding author will on request detail the restrictions and any conditions under which access to some data may be provided.

## Author contribution statement

The NIHR and the MRC had no role in the design or conduct of the study; no role in collection, management, analysis, or interpretation of the data; preparation, review, or approval of the manuscript; and no role in the decision to submit the manuscript for publication in the design, execution or write up of this trial.
