# Supplementary material for: Increased systemic and adipose 11β-HSD1 activity in idiopathic intracranial hypertension
Source: Eur J Endocrinol. 2022 May 18;187(2):323–33. doi: 10.1530/EJE-22-0108 (PMC9346265; doi:10.1530/EJE-22-0108)
Supplement: Supplementary Material [file supplementary_material.pdf]

Metabolite (µg/24hours)	Controls (N=17)	IIH (N=27)	P-value
**Androgen metabolites**			
An	1302.3 (677.9-2153.9)	1469.5 (634.0-2974.0)	0.63
Et	908.3 (595.1-1798.3)	1113.2 (470.8-1726.0)	0.92
11β-OH-An	440.7 (304.2-957.9)	604.9 (268.1-937.8)	0.77
**Androgen precursor metabolites**			
DHEA	124.9 (59.2-355.4)	201.5 (81.3-669.2)	0.26
16α-OH-DHEA	266.8 (112.0-886.5)	534.5 (177.8-1112.0)	0.24
5-PD	120.4 (84.0-203.4)	381.4 (204.8-486.8)	0.0018
5-PT	202.0 (85.0-271.0)	215.1 (114.1-352.1)	0.58
**Mineralocorticoid metabolites**			
THA	83.3 (56.5-126.2)	81.2 (46.2-137.3)	0.83
5α-THA	101.5 (54.6-135.3)	59.0 (42.3-106.1)	0.13
THB	81.1 (68.7-126.7)	98.7 (57.9-162.5)	0.45
5α-THB	247 (130.8-346.4)	180.1 (100.9-349.8)	0.77
**Glucocorticoid precursor metabolites**			
PD	158.5 (83.1-248.6)	164.8 (91.0-448.1)	0.45
17-HP	102.4 (40.5-126.8)	85.2 (41.6-167.6)	0.98
PT	364.6 (256.3-453.1)	340.6 (160.4-622.5)	>0.99
PTONE	6.8 (4.6-11.2)	8.2 (5.6-17.5)	0.26
THS	50.3 (34.1-75.3)	56.5 (36.8-100.9)	0.58
**Total Glucocorticoid metabolites**			
Cortisol (F)	45.8 (28.9-68.2)	71.1 (39.7-90.8)	0.015
THF	1272.6 (864.3-1886.8)	1401.8 (882.5-1657.0)	0.88
5α-THF	1044.8 (697.2-1540.1)	1274.3 (749.0-1699.0)	0.43
α-cortol	330.1 (203.8-479.4)	292.6 (202.2-382.0)	0.56
β-cortol	369.3 (294.6-443.6)	397.9 (300.1-525.7)	0.61
11β-OH-Et	147.0 (79.8-439.5)	254.9 (92.4-424.4)	0.72
Cortisone (E)	74.5 (59.3-107.4)	95.7 (65.9-128.4)	0.33
THE	2545.7 (1835.9.0-3889.3)	2769.6 (1314.0-3489.0)	0.75
α-cortolone	1357.1 (968.7-1868.6)	1200.6 (656.2-1520.0)	0.29
β-cortolone	432.4 (354.0-837.2)	449.0 (282.0-786.9)	0.56
11-oxo-Et	231.3 (124.6-397.8)	276.0 (142.8-406.9)	0.63
**Derivative measurements**			
11βHSD1 (5αTHF+THF/THE)	0.9 (0.7-1.0)	1.1 (0.9-1.4)	0.042
11β-HSD2 (F/E)	0.5 (0.4-0.6)	0.5 (0.4-0.6)	0.88
Total GC metabolites	8317.9 (5361.2-10096.0)	8273.0 (4812.5-10459.5)	0.76

Table S1 Baseline Urinary Steroid Excretion

Urinary steroid profiling on 24-hour urines from control (N=17) and IIH (N=27) patients as measured by GCMS. Abbreviations: An= androsterone; Et= etiocholanolone; 11β-OH-An= 11β-hydroxy-An; DHEA= dehydroepiandrosterone; 16α-OH-DHEA= 16α-hydroxy-DHEA; 5-

PT= Pregnenetriol; 5-PD= Pregnenediol; THA=Tetrahydro-11-dehydrocorticosterone; 5α-THA= 5α- Tetrahydro-11-dehydrocorticosterone; THB=Tetrahydrocorticosterone; 5α-THB= 5α- Tetrahydrocorticosterone; PD= Pregnanediol; 17-HP= 17-hydroxy-pregnanolone; PT= Pregnanetriol; PTONE= Pregnanetriolone; THS= Tetrahydro-11-deoxycortisol; THF= Tetrahydrocortisol; 11β-OH-Et = 11β-hydroxyetiocholanolone; THE= tetrahydrocortisone; 11-OXO-Et= 11-oxo-etiocholanolone; GC= glucocorticoid. Total glucocorticoid excretion is the sum of 5α-THF + THF + THE + cortolones + cortols + cortisol + cortisone. N represents number of patients. Data presented as median (lower quartile-upper quartile). Mann-Whitney-U tests.

Metabolite (µg/24hours)	Baseline		12 months		Δ 12 months from baseline		P-value
	CWM (N=16)	Surgery (N=13)	CWM (N=16)	Surgery (N=13)	CWM (N=16)	Surgery (N=13)	
**Androgen metabolites**							
An	1081.7 (696.2-2138.2)	679.2 (402.9-1850.1)	2288.4 (1003.0-4035)	1320.5 (709.8-2543.8)	908.0 (104.3-2062.5)	516.0 (-810.0-1731.5)	0.39
Et	1665.1 (867.4-2093.3)	1502.9 (510.7-2276.7)	2112.7 (1469.5-3821.3)	1674.9 (865.1-3458.0)	587.0 (-167.8-1550.0)	394.0 (-912.5-2373.5)	0.61
11β-OH-An	612.9 (459.7-1014.4)	395.1 (122.5-956.8)	1725.8 (871.8-1965.76)	1000.8 (524.5-1236.2)	752.0 (210.5-1142.0)	443.0 (-267.5-1020.5)	0.18
**Androgen precursor metabolites**							
DHEA	86.0 (38.4-363.5)	130.0 (79.8-363.9)	76.3 (28.9-257.2)	62.0 (22.4-130.4)	-4.0 (-91.0-15.0)	-82.0 (-253.0-24.0)	0.28
5-PD	43.7 (15.2-122.7)	20.5 (14.4-165.9)	40.7 (19.8-90.6)	38.2 (32.0-91.9)	23.5 (-38-3-58.3)	18.0 (-34.7- 42.7)	0.28
5-PT	287.5 (178.9-431.5)	195.3 (122.8-324.2)	330.5 (218.2-719.5)	398.2 (272.9-(660.1)	28.5 (-57.8-276.25)	178.0 (7.5-511.5)	0.89
**Glucocorticoid precursor metabolites**							
PD	747.7 (312.9-2269.7)	568.7 (269.5-808-1)	982.5 (426.1-1671.4)	1389.3 (815.3-4912.1)	154.0 (-653.8-955.0)	669.0 (239-4061)	0.04
17-HP	189.2 (67.4-352.4)	91.7 (42.9-283.4)	138.1 (110.4-412.7)	345.6 (147.1-647.8)	48.0 (-17.5-143.0)	99.0 (-18.0-484.5)	0.32
PT	741.5 (557.7-1562)	462.9 (281.4-961.9)	875.1 (590.9-1491.8)	701.2 (535.7-1517.9)	57.0 (-141.8-682.5)	233 (265.5-1178.0)	0.81
THS	53.1 (39.9-78.6)	42.8 (26.4-61.8)	77.4 (51.5-100.6)	67.7 (42.3-121.7)	19.5 (-29.3-41.3)	26.9 (-16.5- 74.0)	0.38
**Total Glucocorticoid metabolites**							
Cortisol (F)	62.2 (38.1-76.1)	54.5 (27.1-98.2)	57.3 (44.7-95.4)	71.1 (48.2-85.2)	-5.5 (-35.5-34.8)	14.0 (-38.0- 44.0)	0.82
THF	1799.8 (1251.7-2789.5)	1845.8 (1063.7-3180.9)	2192.8 (1727.1-3452.6)	2243.2 (1174.4-2538.8)	259.5(-656.3-1324.5)	794.0 (-1575.5-1097.5)	0.37
5α-THF	441.5 (225.8-549.3)	198.1 (106.7-491.0)	702.4 (488.2-1226.7)	420.8 (242.4-590.6)	197.5 (69.0-634.3)	221.0 (-321.5-396.5)	0.65

11β-OH-Et	293.7 (132.9-517-1	216.6 (72.3-534.5)	340.9 (65.1-879.8)	248.9 (104.9-538.7)	-0.5 (-296.3-591.0)	-7.0 (-305.5-131.5)	0.62
Cortisone (E)	72.4 (46.8-106.3)	71.8 (36.8-102.7)	93.3 (74.1-172.9)	102.7 (55.6-161.5)	28.0 (-25.3-79.5)	38.0 (-34.5- 91.0)	0.93
THE	2936.4 (2200.1-3758.8)	2891.8 (1249.8-3749.9)	4368.1 (3446.7-6829.9)	3526.6 (2100.5-5618.0)	1227.0 (-463.3-2969.3)	1762.0 (.1337.5-2863.5)	0.87
β-cortolone	780.2 (585.7-1056.7)	537.9 (332.3-786.8)	1694.3 (1220.1-2490.6)	1398.6 (791.3-2064.7)	725.5 (293.0-1531.5)	869.0 (171.0-1274.5)	0.68
**Derivative measurements**							
11βHSD1 (5αTHF+THF/THE)	0.8 (0.7-0.9)	0.9 (0.8-1.1)	0.7 (0.6-0.8)	0.6 (0.5-0.7)	--0.15 (-0.19–0.01)	-0.25 (-0.36–-0.03)	0.01
11β-HSD2 (F/E)	0.8 (0.6-1.0)	0.8 (0.6-1.0)	0.6 (0.5-0.6)	0.6 (0.5-0.8)	-0.24 (-0.29--0.05)	-0.17 (-0.32--0.03)	0.09
Total GC metabolites	9170.7 (7043.9-12640.2)	8403.1 (3693.7-9828.3)	11515.4 (9426.1-18912.1)	11365 (8017-15110.6)	2380 (-1849–6914)	4574 (-3670–10600)	0.82

Table S2 Weight Loss Urinary Steroid Excretion

Urinary steroid profiling via LCMS on 24-hour urines from IIH patients randomised to community weight management (CWM) (N=16) and bariatric surgery (N=13) at baseline and 12 months after baseline. Abbreviations: An= androsterone; Et= etiocholanolone; 11β-OH-An= 11β-hydroxy-An; DHEA= dehydroepiandrosterone;; 5-PT= Pregnenetriol; 5-PD= Pregnenediol; PD= Pregnanediol; 17-HP= 17-hydroxy-pregnanolone; PT= Pregnanetriol; THS= Tetrahydro-11-deoxycortisol; THF= Tetrahydrocortisol; THE= tetrahydrocortisone; Total glucocorticoid excretion is the sum of 5α-THF + THF + THE + cortolones + cortols + cortisol + cortisone; GC= glucocorticoid. N represents number of patients. Data presented as median (lower quartile-upper quartile). Mann-Whitney-U tests between surgery and CWM on Δ 12 month from baseline.
